# INF2 formin variants linked to human inherited kidney disease reprogram the transcriptome, causing mitotic chaos and cell death

**DOI:** 10.1007/s00018-024-05323-y

**Published:** 2024-06-25

**Authors:** Leticia Labat-de-Hoz, Laura Fernández-Martín, Isabel Correas, Miguel A. Alonso

**Affiliations:** 1 Centro de Biología Molecular Severo Ochoa (CBMSO), Consejo Superior de Investigaciones Científicas (CSIC) and Universidad Autónoma de Madrid (UAM), 28049 Madrid, Spain; 2 Department of Molecular Biology, UAM, 28049 Madrid, Spain

**Keywords:** Focal segmental glomerulosclerosis, Charcot-Marie-tooth disease, Formins, Actin, Polarized epithelial cells, Podocytes, Multinucleation, Mitotic catastrophe

## Abstract

Mutations in the human *INF2* gene cause autosomal dominant focal segmental glomerulosclerosis (FSGS)—a condition characterized by podocyte loss, scarring, and subsequent kidney degeneration. To understand INF2-linked pathogenicity, we examined the effect of pathogenic INF2 on renal epithelial cell lines and human primary podocytes. Our study revealed an increased incidence of mitotic cells with surplus microtubule-organizing centers fostering multipolar spindle assembly, leading to nuclear abnormalities, particularly multi-micronucleation. The levels of expression of exogenous pathogenic INF2 were similar to those of endogenous INF2. The aberrant nuclear phenotypes were observed regardless of the expression method used (retrovirus infection or plasmid transfection) or the promoter (LTR or CMV) used, and were absent with exogenous wild type INF2 expression. This indicates that the effect of pathogenic INF2 is not due to overexpression or experimental cell manipulation, but instead to the intrinsic properties of pathogenic INF2. Inactivation of the INF2 catalytic domain prevented aberrant nuclei formation. Pathogenic INF2 triggered the translocation of the transcriptional cofactor MRTF into the nucleus. RNA sequencing revealed a profound alteration in the transcriptome that could be primarily attributed to the sustained activation of the MRTF-SRF transcriptional complex. Cells eventually underwent mitotic catastrophe and death. Reducing MRTF-SRF activation mitigated multi-micronucleation, reducing the extent of cell death. Our results, if validated in animal models, could provide insights into the mechanism driving glomerular degeneration in INF2-linked FSGS and may suggest potential therapeutic strategies for impeding FSGS progression.

**Supplementary Information:**

The online version contains supplementary material available at 10.1007/s00018-024-05323-y.

## Introduction

Focal segmental glomerulosclerosis (FSGS) is a morphological/histological pattern of kidney injury characterized by podocyte loss and the presence of scarring in some, but not all, glomeruli, and in which only a portion of the glomerulus is affected. This alteration causes the deterioration of the structure of the glomerular filtration barrier, compromising its blood filtration capacity. FSGS initially manifests as proteinuria, frequently progresses to chronic kidney disease, and, in the most severe cases, leads to end-stage kidney disease [1]. FSGS has genetic and non-genetic causes [2]. Among the former, mutations of the *INF2* gene are the main cause of autosomal dominant FSGS, accounting for up to 17% of familial cases and 1% of sporadic cases [3–5].

The INF2 protein belongs to the formin family, which in humans consists of 15 members whose function is to polymerize monomeric globular actin (G-actin) into linear filaments (F-actin) [6–8]. INF2 activity, as with most formins, is regulated by the association of the diaphanous inhibitory domain (DID) in the molecule’s N-terminal half with the diaphanous autoinhibitory domain (DAD) in the C-terminal region. The core region of the molecule hosts the formin homology 1 (FH1) domain, which is responsible for binding profilin bound to G-actin, and the catalytic FH2 domain, which uses the G-actin provided by profilin to create F-actin. The intramolecular DID-DAD association hides these domains, rendering INF2 inactive [6, 9]. The DID-DAD interaction in INF2 is weaker than that of mouse *Dia* (*mDia*) formins [10], and requires reinforcement through a complex formed by cyclase-associated protein (CAP) and lysine-acetylated actin (KAc-actin) [11]. Specific Rho GTPase binding in the GTP-loaded active state disrupts the inhibitory DID-DAD interaction in *mDia*1-3 and other formins, liberating the FH2 domain [9]. However, INF2 activation involves calmodulin (CaM) binding to a helical peptide motif in the N-terminal extension preceding the DID [12, 13]. In addition to cytoplasmic F-actin formation, INF2 is implicated in nuclear F-actin assembly [14], microtubule binding and stabilization [15, 16], vesicular transport [17, 18], mitochondrial fission [19, 20], and cell extrusion [21].

More than 60 pathogenic mutations have been identified in INF2, all of them arising within the DID. Depending on the specific mutation, they lead to FSGS alone [4] or co-occurring with Charcot-Marie-Tooth (CMT) disease [22], which is a degenerative condition affecting the peripheral nerves [23]. Mutations causing FSGS alone are distributed across the DID, while those associated with both FSGS and CMT are mostly concentrated in the N-terminal half of the DID [24]*.* Biochemical analyses indicate that the pathogenic mutations of INF2 disrupt the intramolecular DID-DAD association [25], hinder the regulatory intermolecular interaction between the INF2 DID and the *mDia*1-3 DAD [10], and greatly reduce INF2 binding to Ca^2+^/CaM [26] and to the CAP-KAc-actin complex [27]. Consequently, pathogenic INF2 becomes deregulated and displays constitutive high actin polymerization activity [11, 26]. Despite significant advances in our understanding of normal and pathogenic INF2, the precise causes of the severe cellular impact leading to progressive glomerular deterioration in FSGS remain elusive.

To understand the mechanism underpinning the detrimental effect of pathogenic INF2, in this work we have investigated the effect of its expression on the cell by examining specific cell lines and human primary podocytes. Cells exhibited an increase in the number of microtubule-organizing centers (MTOCs) during mitosis. These surplus MTOCs, which arise from centrosome fragmentation, orchestrated the assembly of multiple mitotic spindles, disrupting the precise alignment of chromosomes on the metaphase plate. Consequently, mitoses ended with cells displaying diverse morphological nuclear anomalies, prominently featuring multiple micronuclei, ultimately leading to cell death and detachment from the substrate. Notably, we found that mutating the catalytic domain of INF2, rendering it inactive, prevented the formation of aberrant nuclei. Moreover, pathogenic INF2 prompted the translocation of the transcriptional cofactor myocardin-related transcription factor (MRTF) [28] into the nucleus, resulting in a profound alteration in the transcriptome. Interference with MRTF translocation produced a notable reduction in the frequency of nuclear abnormalities and protected the cells from undergoing death. Our findings could help identify the path leading to glomerular degeneration in INF2-linked FSGS, potentially paving the way for innovative therapeutic strategies that impede progression from initial glomerular damage to advanced kidney deterioration.

## Results

### Pathogenic INF2 expression induces cell toxicity

INF2 is expressed as two isoforms, INF2-1 (also known as INF2-CAAX) and INF2-2 (also known as INF2-non-CAAX). INF2-1 (hereafter denoted as INF2 except when compared with INF2-2), has a consensus CAAX prenylation box at the C-terminal end, undergoes farnesylation, and localizes to the endoplasmic reticulum (ER) [29]. Conversely, INF2-2 concludes with an alternative sequence and is cytosolic [18, 29, 30]. To assess the expression levels of pathogenic INF2 variants that cause FSGS in comparison to wild-type (wt) INF2 we used a chimera of the fluorescent Cherry protein fused to the N-terminus of the entire INF2 R218Q molecule. The INF2 R218Q variant was chosen because it is the most frequently occurring INF2 pathogenic variant [24]. Distinct pathogenic variants can affect the cells to varying degrees, reflecting the ranges of glomerular damage observed in patients and of the timing of FSGS onset [24]. The onset of end-stage renal disease in patients with the INF2 L76P mutation, which causes solely FSGS, occurs over a range of ages of 28–70 years. In contrast, the range starts and ends at earlier ages for the other familial mutations analyzed in the same study [31]. This suggested us that the L76P mutation might have a milder effect. With this rationale, we chose INF2 L76P for comparative purposes in some experiments. As a model renal cell system, we initially adopted MDCK cells, a non-transformed cell line derived from canine distal tubules, which are considered a paradigm of normal polarized epithelial cells [32]. Unless otherwise indicated, recombinant retrovirus infection was used to facilitate e expression of exogenous INF2 proteins throughout this work.

**Fig. 1:**
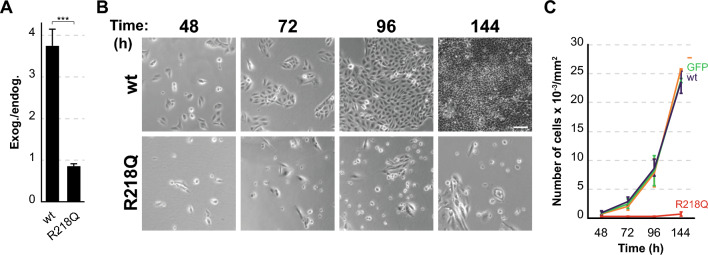
Pathogenic INF2 R218Q induces toxicity in MDCK cells. **A** Graph displaying exogenous wt INF2 and INF2 R218Q protein levels relative to endogenous INF2 after 48 h of expression. The percentages of positive cells were considered for the estimations. ***, *p* < 0.001. **B** Cells expressing GFP or GFP fused to wt INF2 or INF2 R218Q were sorted after 24 h of expression. An equal number of untreated cells and GFP-sorted cells were plated, and after 24 h, cell fields were imaged to determine the number of cells attached to the substrate at the indicated times. Representative fields of cells expressing wt INF2 and INF2 R218Q are shown. Scale bar, 40 μm. **C** The graph depicts the number of cells attached to the substrate for each condition. More than 200 cells from at least three different cell fields were analyzed for each experimental condition. Three independent experiments were performed in **A**, **C**

The peak expression of wt, R218Q and L76P INF2 proteins occurred at 48 h (Fig. S1A). At this time, accounting for the percentage of cells expressing exogenous INF2 in each case, we found that the expression levels of INF2 R218Q were comparable to those of endogenous INF2 and that, consistent with previous findings [26], the expression of exogenous wt INF2 was significantly higher than that of INF2 R218Q (Figs. 1A and S1B). Quantitative PCR analysis revealed a five-fold lower level of INF2 R218Q transcripts relative to wt INF2 (Fig. S1C). Consequently, the lower level of expression of INF2 R218Q can be primarily attributed to the low levels of its mRNA.

To understand the harmful nature of pathogenic INF2, we compared the survival of cells expressing either wt INF2 or INF2 R218Q. Following 24 h of expression, cells positive for GFP alone or GFP fused to wt INF2 and INF2 R218Q were sorted to ensure homogeneous cell populations, and an equal number of each type of cell were subsequently plated and cultured for 24 h. After that, cells were monitored using bright-field microscopy over a span of 4 days. This analysis revealed a stark contrast: while GFP and wt INF2-expressing cells exhibited continuous proliferation, those expressing INF2 R218Q, instead, underwent cell death and detachment from the substrate from the onset of the analysis (Fig. 1B, C). This outcome indicates that pathogenic INF2, which was expressed at levels comparable to those endogenous INF2 (Fig. 1A), has a toxic nature. In contrast, wt INF2, which was overexpressed about fourfold relative to endogenous INF2 (Fig. 1A), was innocuous.

### Pathogenic INF2 induces distinct nuclear abnormalities

**Fig. 2:**
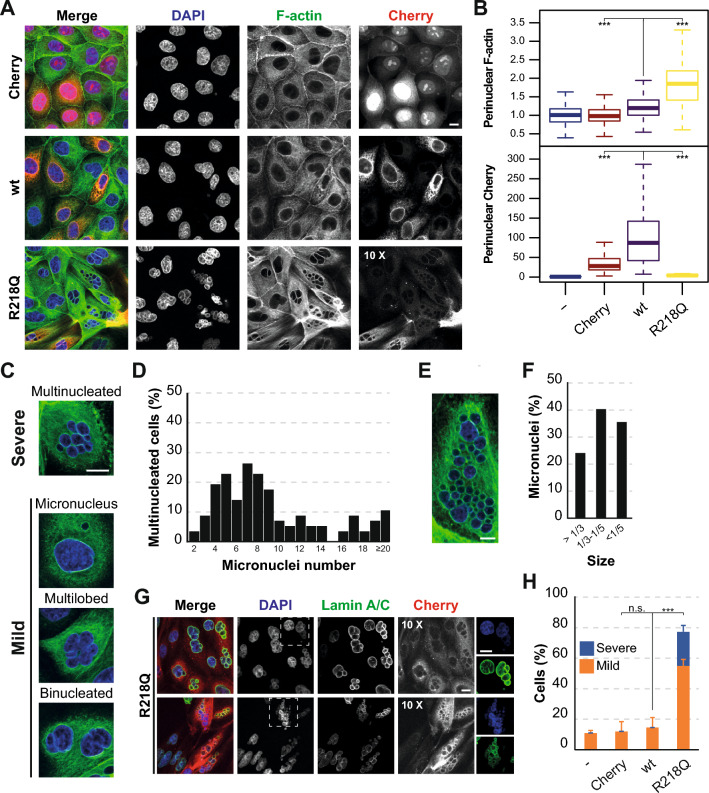
Nuclear morphology of INF2 R218Q MDCK cells. **A** F-actin staining of Cherry, wt INF2 and INF2 R218Q cells. The contrast in the Cherry channel was increased as indicated to visualize the cells expressing INF2 R218Q. **B** Box plots depicting staining intensity of F-actin and the indicated Cherry proteins at the perinuclear region of three equatorial planes relative to that of untreated cells. The perinuclear Cherry content was normalized to the background intensity value of untreated cells. At least 150 cells were analyzed for each experimental condition in three independent experiments. **C** Classification of abnormal classes of nuclear morphologies observed. **D** Percentage of multi-micronucleated cells with the indicated numbers of micronuclei. **E** Image of an INF2 R218Q cell with over 40 micronuclei. **F** Percentage of micronuclei with size > 1/3, 1/3–1/5 and < 1/5 of the mean nuclear diameter of a normal mononucleated cell. 114 multi-micronucleated cells were analyzed in **D**, **F**. **G** Images of INF2 R218Q cells stained for lamin A/C. **H** Percentage of untreated, Cherry, wt INF2 and R218Q cells with mild or severe nuclear phenotypes. Over 450 cells were analyzed for each experimental condition in three independent experiments. Nuclei were visualized using DAPI. Scale bars, 10 μm. n.s., not significant; ***, *p* < 0.001

To identify alterations associated with the detrimental effect of pathogenic INF2 on cells, cells positive for Cherry alone (Cherry cells) or fused to wt INF2 (wt INF2 cells) and INF2 R218Q (INF2 R218Q cells) were fixed and stained for F-actin. Unless otherwise indicated, this and the other experiments reported were conducted with unsorted cell populations after 48 h of exogenous protein expression. Consistent with the analysis of the expression levels by immunoblotting (Figs. 1A and S1B), confocal microscopic analysis revealed markedly lower INF2 R218Q signal intensity compared with wt INF2, but contrast adjustment allowed unambiguous identification of positive cells (Fig. 2A). Quantitative assessment of three equatorial planes in the perinuclear region revealed higher levels of F-actin in INF2 R218Q cells than in the three types of control cell used (untreated, Cherry, and wt INF2 cells) (Fig. 2B), in line with previous studies indicating deregulated activity in pathogenic INF2 [11, 26]. Further scrutiny revealed distinct classes of nuclear morphological abnormalities in INF2 R218Q cells. These abnormalities encompassed multiple micronuclei, a single micronucleus, a multilobed nucleus, and, occasionally, two seemingly normal nuclei (Fig. 2C). The number of micronuclei in INF2 R218Q cells ranged from 2 to more than 20 per cell, with an average of 9.5 ± 6.0 (Fig. 2D, E), 75% of them with a diameter less than one-third of the mean diameter (17.5 ± 2.3 μm) of a normal nucleus (Fig. 2F). Notably, all micronuclei exhibited a complete lamina (Fig. 2G). The presence of multiple micronuclei was designed as a “severe” phenotype, since cells with this nuclear characteristic normally do not to support new cycles of cell division [33]. The rest of the nuclear abnormalities were grouped as a “mild” phenotype since cells occasionally manage to overcome these defects in the subsequent cell cycle [33]. The mild nuclear abnormalities sometimes appeared in combination, such as cells with a micronucleus alongside with a nucleus with multilobed morphology or a binucleated cell with similar multilobed nuclei. For the mild nuclear phenotype, the presence of a multilobed nucleus or a single micronucleus was much more frequent than binucleation (Fig. S2A). Approximately 80% of INF2 R218Q cells exhibited nuclear abnormalities, 25% being severe and 55% of them mild. In contrast, no severe abnormalities were found, and only 12–15% displayed mild nuclear phenotypes, among the three types of control cell (Fig. 2H).

The abnormal INF2 R218Q cells were enlarged and less circular than control cells (Fig. S2B, C). Untagged and GFP-tagged INF2 R218Q both exhibited the same classes of nuclear abnormalities as Cherry-tagged INF2 R218Q (Fig. S2D). This result indicates that the tag does not contribute to the observed abnormalities. Furthermore, transfection of a DNA construct expressing Cherry-INF2 R218Q under the late CMV promoter also resulted in nuclear aberrations and cell death (Fig. S2E, F). In summary, the effect of pathogenic INF2 on nuclear morphology was not due to overexpression (Fig. 1A) and was independent of the tag (Fig. S2D), the promoter (LTR or CMV) (Figs. 2H and S2E), and the expression system (retroviral infection or plasmid transfection) used (Figs. 2H and S2E). Additionally, cell death induced by pathogenic INF2 was independent of whether the cells were sorted (Fig. 1B, C) or not (Fig. S2F).

### Other pathogenic variants of INF2 also cause nuclear abnormalities, not just R218Q

As previously reported [12], compared with control cells, INF2 KO cells had a scarce cytosolic F-actin content and lacked the ring of F-actin that surrounds the nucleus (Fig. S2G). INF2 R218Q induced a clear increase of F-actin over that of wt INF2 in INF2 KO cells (Fig. S2G). The effect of the expression of INF2-1 R218Q on the nuclear morphology of INF2 KO cells yielded results mirroring those obtained from normal cells (Fig. S2H), indicating that it does not require the expression of endogenous INF2 to induce nuclear abnormalities. INF2-2 R218Q also altered nuclear morphology, although its effect was less pronounced (Fig. S2H). Similar to INF2 R218Q, other pathogenic INF2 variants—such as those exclusively associated with FSGS (L57P, L76P, L165P), those linked to a combination of FSGS and CMT (L128P), and those responsible for FSGS in certain patients and FSGS and CMT in others (L77P, R106P) [24]—also generated nuclear defects, INF2 L76P having the mildest effect (Fig. S2I)*.* As a control, we observed that three of the presumably benign INF2 variants present in the human population—V194M, R212C, and R261G—behaved similarly to wt INF2 (Fig. S2J).

### Pathogenic INF2 induces nuclear abnormalities in specific cell lines

**Fig. 3:**
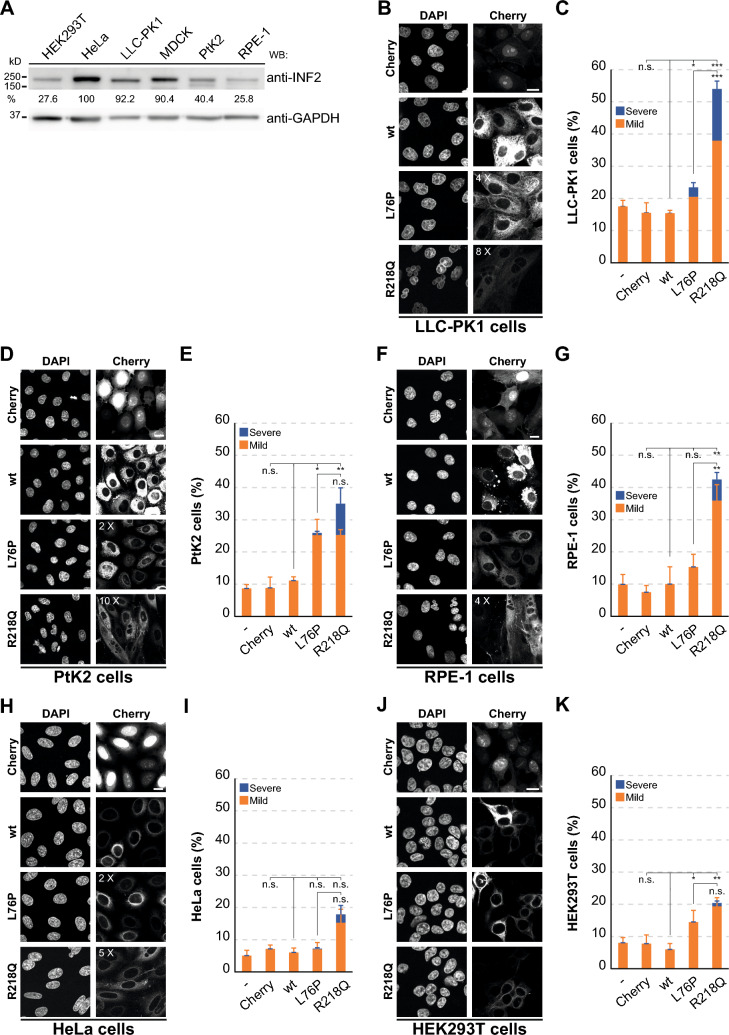
Nuclear phenotype in a panel of cell lines expressing pathogenic INF2. **A** Endogenous INF2 expression analyzed by immunoblotting with anti-INF2 antibodies in the indicated cell lines, using GAPDH as a loading control. The numbers show the percentage of INF2 levels in the different cell lines relative to those in HeLa cells, which had the highest level of expression. **B**–**K** Images of an equatorial plane of the indicated cell lines expressing Cherry alone or Cherry fused to wt, L76P, and R218Q INF2 (**B**, **D**, **F**, **H**, **J**), and quantification of the percentage of cells displaying mild or severe nuclear phenotypes (**C**, **E**, **G**, **I**, **K**). More than 450 cells were analyzed per cell line and condition; three independent experiments. The contrast in the Cherry channel was increased as indicated to identify the cells expressing pathogenic INF2. Nuclei were visualized using DAPI. Scale bars, 15 μm. n.s., not significant; *, *p* < 0.05; **, *p* < 0.01; ***, *p* < 0.001

To explore whether pathogenic INF2 causes similar nuclear defects in other cell lines, we examined a diverse panel encompassing both non-transformed (LLC-PK1, PtK2 and RPE-1) and transformed (HeLa and HEK293T) epithelial cell lines. Given the ubiquitous expression of INF2, all cell lines expressed endogenous INF2, albeit at different levels (Fig. 3A). In non-transformed epithelial cells both INF2 L76P and INF2 R218Q both produced alterations in nuclear morphology to different degrees (Fig. 3B–G), although INF2 R218Q consistently showed a more pronounced effect. In contrast, no significant alterations in nuclear morphology were observed in HeLa cells and changes were relatively moderate in HEK293T cells (Fig. 3H–K). These findings indicate that pathogenic INF2-induced abnormal nuclei form specifically in non-transformed cells, highlighting a distinct response from that of transformed cells.

In summary, the expression of pathogenic INF2 variants in non-transformed cells leads to diverse classes of nuclear abnormalities, of which multi-micronucleation emerges as the most severe anomaly.

### Pathogenic INF2 induces mitotic chaos

**Fig. 4:**
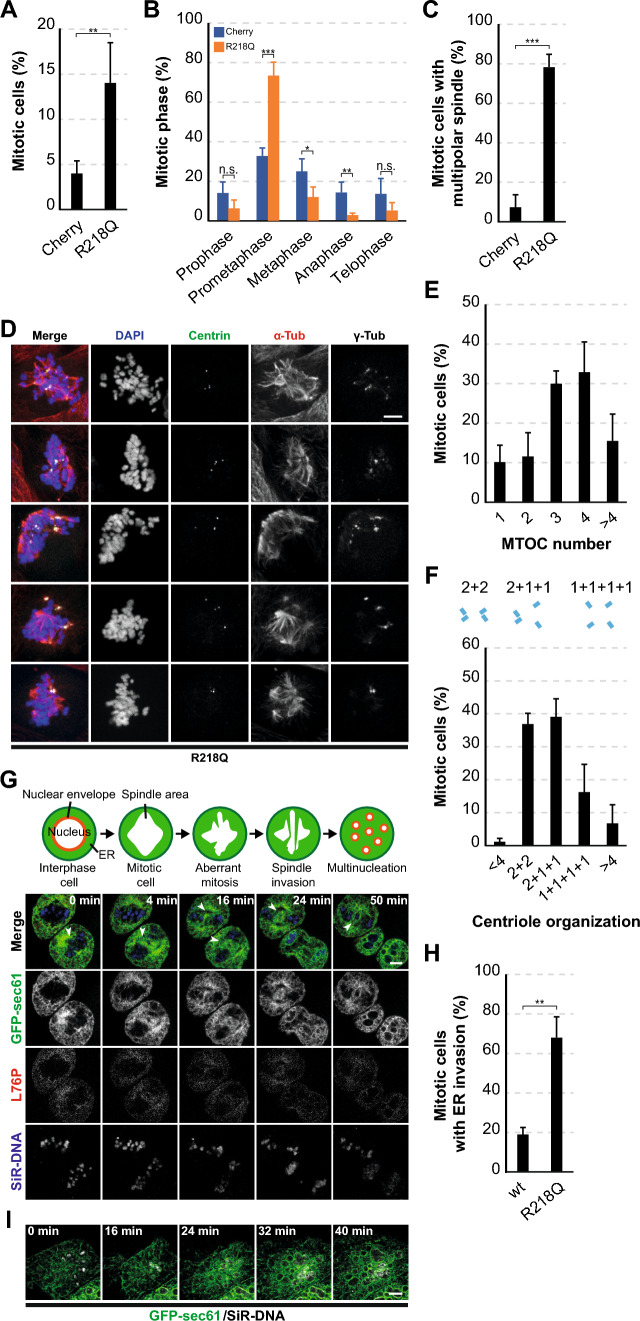
INF2 R218Q induces the formation of multipolar spindles in MDCK cells. **A** Percentage of Cherry and INF2 R218Q cells in mitosis after 48 h of expression. More than 1000 cells were analyzed for each experimental condition in five independent experiments. **B** Distribution of Cherry and INF2 R218Q cells across different phases of the cell cycle. More than 200 cells were analyzed for each experimental condition, four independent experiments. **C** Percentage of mitotic Cherry and INF2 R218Q cells displaying multipolar spindles. 68 Cherry cells and 152 INF2 R218Q cells were examined; four independent experiments. **D** Images of INF2 R218Q mitotic cells stained for centrin, α-tubulin and γ-tubulin. Nuclei were visualized using DAPI. **E** Classification of INF2 R218Q mitotic cells based on the number of MTOCs expressed as percentage of total cells. MTOCs were identified by γ-tubulin and α-tubulin staining. Over 200 cells were examined; four independent experiments. **F** Top: Schematic of centriole arrangements. Bottom: centriole arrangement, as visualized with centrin, in INF2 R218Q mitotic cells categorized as clustered in two centrosomes (2 + 2), disengaged in one (2 + 1 + 1) or both (1 + 1 + 1 + 1) centrosomes, or with an abnormal number of centrioles, presented as a percentage of total cells. More than 170 cells were examined; three independent experiments. **G** Top panel: schematic of ER invasion of the spindle space in cells expressing pathogenic INF2. Bottom panels: INF2 L76P cells stably expressing GFP-sec61 and stained with SiR-DNA analyzed by videomicroscopy during mitosis. The arrowheads indicate regions of the mitotic spindle invaded by ER membranes. **H** The graph shows the percentage of wt INF2 and INF2 R218Q cells with the spindle space invaded by ER membranes. More than 80 cells were analyzed; three independent experiments. **I** Videomicroscopic analysis of INF2 R218Q cells stably expressing GFP-sec61 and stained with SiR-DNA during formation of multiple micronuclei. Scale bars, 5 μm. n.s., not significant; *, *p* < 0.05; **, *p* < 0.01; ***, *p* < 0.001

The presence of nuclear abnormalities strongly indicates underlying mitotic problems [34]. Notably, a significant accumulation of mitotic cells was evident in INF2 R218 cells compared with Cherry cells (Fig. 4A). Upon categorization into different mitotic phases based on chromosome arrangement, we concluded that INF2 R218 cells accumulated in prometaphase (Fig. 4B), because their chromosomes were not properly aligned at the metaphase plate, often appearing scattered throughout the cytoplasm [34]. Staining for α-tubulin revealed that, unlike normal cells, around 80% of INF2 R218Q cells assembled multipolar spindles (Figs. 4C, D and S3A) instead of bipolar spindles, as occurs in normal cells. Quantification analysis showed that most mitotic INF2 R218Q cells had 3–4 MTOCs (Fig. 4E). The increased frequency of MTOCs might stem from supernumerary centrioles or from centrosome fragmentation, a process involving centriole disengagement and dispersal of pericentriolar material that results in the formation of centriolar and acentriolar MTOCs, respectively [35]. Staining for centrin revealed a substantial proportion of mitotic INF2 R218Q cells with centrioles disengaged from one (39%) or both (16%) centrosomes (Fig. 4D, F). The sum of these percentages (55%) is lower than the percentage of INF2 R218Q cells with supernumerary MTOCs (78%), suggesting the existence of centriolar and acentriolar MTOCs. Hence, centrosome fragmentation, rather than the generation of supernumerary centrioles, causes the formation of multipolar spindles in INF2 R218Q cells.

Similar to normal mitosis [36], we noted an initial exclusion of ER membranes from the spindle zone in INF2 R218Q cells, as revealed using the GFP-sec61β ER marker. However, as the multipolar spindle developed, the ER, but not F-actin, invaded the spindle space (Fig. 4G and Videos 1 and 2). ER intrusion occurred in around 70% INF2 R218Q cells (Fig. 4H) and resulted in the formation of multiple micronuclei (Fig. 4I and Video 3). No substantial alterations were detected in the levels of α-tubulin or microtubule posttranslational modifications in INF2 R218Q cells (Fig. S3B). Similar to INF2 R218Q MDCK cells, INF2 R218Q PtK2 cells exhibited a heightened proportion of mitotic cells in prometaphase showing multipolar spindles (Fig. S3C–E).

In summary, observations from Figs. 4 and S3 highlight the centrosome fragmentation in INF2 R218Q cells, leading to the formation of multipolar spindles. These aberrant spindles hinder the proper alignment of chromosomes on the metaphase plate and permit the invasion of the spindle space by the ER. Consequently, ER intrusion results in the packaging of chromosomes into multiple micronuclei.

### INF2 R18Q cells experience mitotic catastrophe

**Fig. 5:**
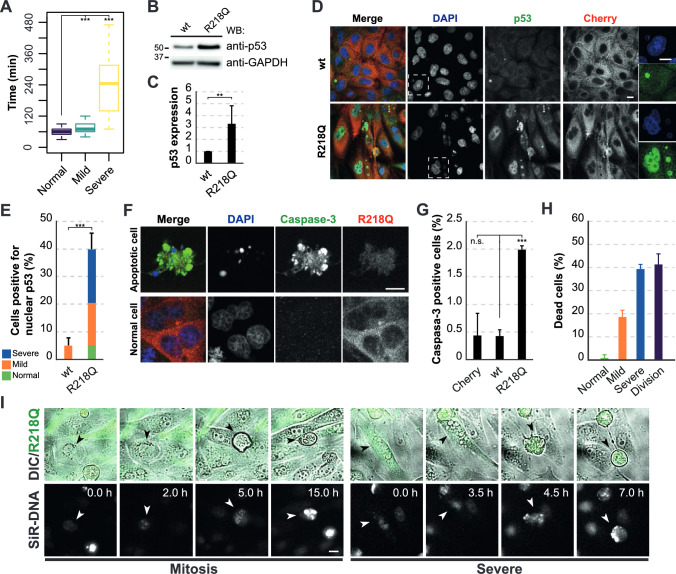
INF2 R218Q induces nuclear accumulation of p53 and activation of caspase-3 in MDCK cells. **A** The graph depicts the time from prophase initiation to the end of cell division, as determined by time-lapse analysis, in control cells (n = 63) and in cells expressing pathogenic INF2 ending with mild (n = 48 cells) and severe (n = 36 cells) nuclear defects; three independent experiments were performed. MDCK cells stably expressing H2B-GFP were used in this experiment. **B** Immunoblot analysis of p53 in wt INF2 and INF2 R218Q cells. GAPDH was used as loading control. **C** Quantification of p53 levels in wt INF2 and R218Q cells. Five independent experiments were performed. **D** Image of p53 staining in an equatorial plane of wt INF2 and INF2 R218 cells. Cells were stained with anti-Cherry antibodies to help visualization of the exogenous proteins. **E** Percentage of cells with nuclear p53, considering staining in the main nucleus or micronuclei as positive. Over 350 cells were examined; three independent experiments. **F** Images of cleaved caspase-3 staining in apoptotic INF2 R218Q cells (top) and normal cells (bottom). Nuclei were visualized with DAPI. **G** Percentage of Cherry, wt INF2 and INF2 R218Q cells positive for cleaved caspase-3 after 48 h of expression. More than 700 cells were examined per experimental condition; three independent experiments. **H** The percentage of cells with normal, mild or severe nuclear morphology or in mitosis that died between 55 and 72 h of INF2 R218Q expression was determined by time-lapse microscopy in cells labeled with SiR-DNA. 130 cells were analyzed; three independent experiments. **I** Videomicroscopic analysis of a mitotic (left panels) and an INF2 R218Q cell with a severe phenotype (right panels) undergoing death and detachment from the substrate. Arrowheads indicate the dying cell. Nuclei were stained with SiR-DNA. DIC, differential interference contrast microscopy. Scale bars, 10 μm (**D**, **F**), 250 μm (**I**). n.s., not significant; **, *p* < 0.01; ***, *p* < 0.001

Mitotic catastrophe is defined as an antiproliferative mechanism that senses and responds to mitotic failure by inducing cell death or senescence. It arises during abnormal mitosis or, if the cells complete a flawed mitosis, during the subsequent cell cycle [37]. Mitotic catastrophe is typical of enlarged cells exhibiting multinucleation, as seen in cells expressing pathogenic INF2 (Figs. 2H and S2B), and whose mitosis is accompanied by some degree of mitotic arrest [37]. To ascertain if cells expressing pathogenic INF2 possess the latter characteristic, we measured the time taken to progress from nuclear envelope breakdown to the completion of telophase using time-lapse microscopy. Our findings revealed a substantial increase in mitosis duration in pathogenic INF2-expressing cells that was notably longer in cells ending with severe nuclear abnormalities than in those with mild aberrations (Fig. 5A).

The p53 tumor suppressor acts to produce sequence-specific DNA transcription, which is crucial for safeguarding genomic stability in non-transformed cells [38]. Typically, p53 has a short half-life, is maintained at low levels, and is mostly cytosolic in unstressed cells. However, in response to mitotic or genomic stress, p53 undergoes stabilization and accumulates in the nucleus. Here it orchestrates transcriptional programs involved in DNA repair, cell cycle arrest, senescence and apoptosis [39–41]. To investigate the mechanism of cell death caused by pathogenic INF2, we examined the levels of p53 and its subcellular distribution. p53 levels were upregulated approximately threefold in INF2 R218Q cells relative to wt INF2 cells (Fig. 5B, C). Notably, in INF2 R218Q cells, p53 predominantly localized within the nucleus, contrasting with its cytoplasmic distribution in wt INF2 cells (Fig. 5D, E).

Caspase-3 is an effector caspase that is pivotal in the later stages of apoptosis [42]. After 48 h of INF2 R218Q expression, approximately 2% of cells were positive for cleaved caspase-3, a significantly higher proportion than in wt INF2 and Cherry cells, which served as control cells (Fig. 5F, G). It is important to note that this analysis represents a static snapshot of caspase-3-positive cells at a given moment. Considering the continuous emergence and elimination of apoptotic cells from the cell monolayer, our findings suggest that INF2 R218Q cells undergo programmed cell death, probably apoptosis, as the mode of cell death. Videomicroscopic analysis revealed that roughly 80% of cells perished during mitosis or as multi-micronucleated cells, while the remaining cells exhibited apparently milder nuclear alterations at the time of cell death (Fig. 5H). Furthermore, this analysis depicted instances illustrating detachment of dead cells from the substrate (Fig. 5I and Video 4).

No induction of p21, a cyclin-dependent kinase inhibitor that is crucial for cell cycle arrest and senescence [43], expression was detected in INF2 R218Q cells (Fig. S4A). Additionally, INF2 R218Q cells were positive for the nuclear Ki67 antigen (Fig. S4B, C), a proliferation marker typically absent from quiescent cells [44], further indicating the absence of cell senescence.

In conclusion, INF2 R218Q cells experience mitotic catastrophe during or after mitosis, leading to cell death and subsequent detachment from the substrate.

### The development of nuclear abnormalities induced by pathogenic INF2 is independent of CaM binding

**Fig. 6:**
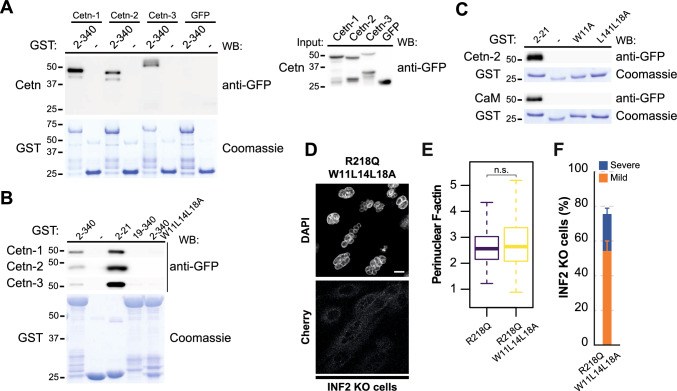
The formation of nuclear abnormalities is independent of centrin or calmodulin binding to pathogenic INF2. **A** GST fused to the 2-340 INF2 fragment (GST-INF2 2-340), which encompasses the INF2 DID, was used in pull-down experiments with GFP-centrin (Cetn)-1, -2 and -3 or GFP alone (left panel). The input of the four GFP proteins used is shown (right panel). **B** Pull-down of various INF2 fragments (2-340, 2-21, 19-340, and 2-340 with the W11L14L18A mutation) with GFP-Cent-1, -2 and -3. **C** Pull-down of the 2-21 INF2 fragment with the indicated mutations in residues W11, L14 and L18 with GFP-Cent-2 or GFP-CaM. Purified GST proteins were stained with Coomassie blue to control for the amount of GST protein used in (**A**–**C**). **D** Image of an equatorial plane of INF2 KO MDCK cells expressing INF2 R218Q W11L14L18A. Nuclei were visualized with DAPI. Scale bar, 10 μm. **E** Perinuclear F-actin levels in INF2 R218Q and INF2 R218Q W11L14L18A cells. More than 150 cells were examined; three independent experiments. **F** Percentage of INF2 R218Q W11L14L18A cells displaying an abnormal nuclear phenotype. More than 300 cells were analyzed; three independent experiments. n.s., not significant

Ca^2+^/CaM binds to INF2 and initiates its actin polymerization activity [13]. The EF-hands in the C-terminal lobe of CaM specifically interacts with a unique CaM-binding site in the first of the two α-helices situated in the short N-terminal extension adjacent to the DID [12]. Human centrins 1-3 constitute a family of Ca^2+^ sensors that, similar to CaM, encompass N-terminal and the C-terminal EF-hands [45]. Notably, centrin-2 binds targets with helical sequences featuring a WxxLxxxL (where x is a spacer amino acid) motif, which coincides with the INF2 Ca^2+^/CaM-binding motif [12, 46]. To determine whether centrins bind to INF2, we performed pull-down assays utilizing GST fusions of amino acids 2-340 INF2 and extracts of HEK293T cells expressing GFP fused to individual centrins. All three centrins exhibited binding (Fig. 6A). Similar to CaM, centrins bound to the 2-340 INF2 fragment and to the sequence encompassing residues 2-21 that house the CaM-binding site, but they did not bind to the 19-340 fragment or the 2-340 INF2 fragment with the triple W11L14L18A mutation (Fig. 6B), which disrupts CaM binding [12]. Mutation of the W11 residue to Ala or the double mutation of residues L14 and L18 to Ala abolished centrin binding (Fig. 6C), indicating that W11 and L14L18 are both essential for the binding. The levels of perinuclear actin and the percentage of abnormal nuclei induced by INF2 R218Q were unaffected by the W11L14L18A mutation (Fig. 6D–F). This result implies that the generation of abnormal nuclei by pathogenic INF2 is independent of CaM and centrin binding to the N-terminal extension of INF2.

### Pathogenic INF2-mediated actin polymerization is essential for generating nuclear abnormalities

**Fig. 7:**
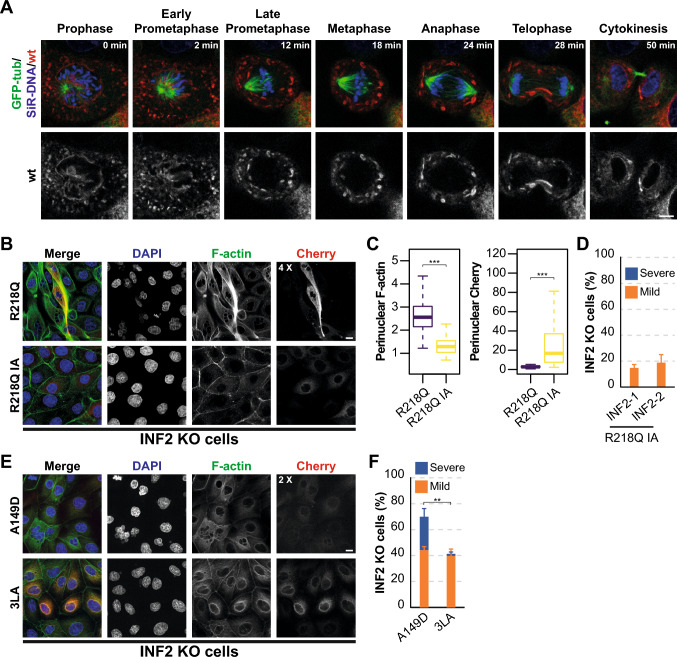
INF2 R218Q requires integrity of its actin polymerization activity to produce nuclear abnormalities. **A** Dynamics of wt INF2 during mitosis in MDCK cells expressing GFP-tubulin. Cells were stained with SiR-DNA. **B** Images of INF2 KO MDCK cells expressing INF2 R218Q and R218Q IA. (**C**) Box plots showing the intensity of the perinuclear F-actin and Cherry corresponding to INF2-1 R218Q and INF2-1 R218Q IA MDCK KO cells. More than 150 cells were examined; three independent experiments. **D** Percentage of MDCK KO cells expressing INF2-1 R218Q IA and INF2-2 R218Q IA exhibiting mild or severe nuclear phenotypes. More than 350 cells were examined; three independent experiments. **E** Images of an equatorial plane of INF2 KO MDCK cells expressing INF2 A149D and INF2 3LA. **F** Percentage of cells expressing INF2 A149D and INF2 3LA displaying mild or severe nuclear phenotypes. More than 300 cells were examined across three independent experiments. The contrast in the Cherry channel was increased as indicated to identify the cells expressing mutant INF2. Nuclei were visualized with DAPI. Scale bars, 5 μm (**A**) and 10 μm (**B**, **E**). **, *p* < 0.01; ***, *p* < 0.001

DIAPH formins are known to localize to key structures for karyokinesis and cytokinesis, and play important roles in these processes [47–50]*.* To determine whether this is also the case for INF2, we used videomicroscopy to track the dynamics of wt INF2 in dividing cells. wt INF2 was present in clusters at the ER and was excluded from the mitotic apparatus and spindle space (Fig. 7A and Video 5). INF2 KO cells are known to divide normally [13, 51, 52], indicating that INF2 is not essential for mitosis. Therefore, the effect of pathogenic INF2 on mitosis is probably indirect.

To investigate whether pathogenic INF2 relies on its actin polymerization activity to promote abnormal nuclear formation, we introduced the I643A (IA) mutation, which is known to block INF2 activity [53]. As a control, we observed that F-actin levels at the perinuclear region were lower in INF2 R218Q IA cells compared with INF2 R218Q cells, despite the higher expression of the double mutant (Fig. 7B, C). Remarkably, the IA mutation effectively halted the nuclear morphology changes induced by both INF2-1 R218Q and INF2-2 R218Q (Fig. 7D).

The A149D mutation was engineered through in silico design to render INF2 constitutively active [54]. The triple L1010L1011Leu1020 mutation to Ala mutation (3LA) disrupts the WH2 motif within the DAD [55], potentially impeding its interaction with KAc-actin and thereby compromising the inhibitory role of the KAc-CAP complex [27]. It is of note that both the non-naturally occurring A149D and 3LA INF2 mutants mirrored the effect of pathogenic INF2 on nuclear morphology (Fig. 7E, F). The comparatively milder effects of INF2 3LA on F-actin levels and nuclear morphology might stem from incomplete deregulation of its activity relative to INF2 A149D.

In conclusion, the findings depicted in Fig. 7 underscore the crucial role of deregulated actin polymerization of INF2 in generating nuclear abnormalities.

### Pathogenic INF2 triggers activation of the MRTF-SRF transcriptional complex

**Fig. 8:**
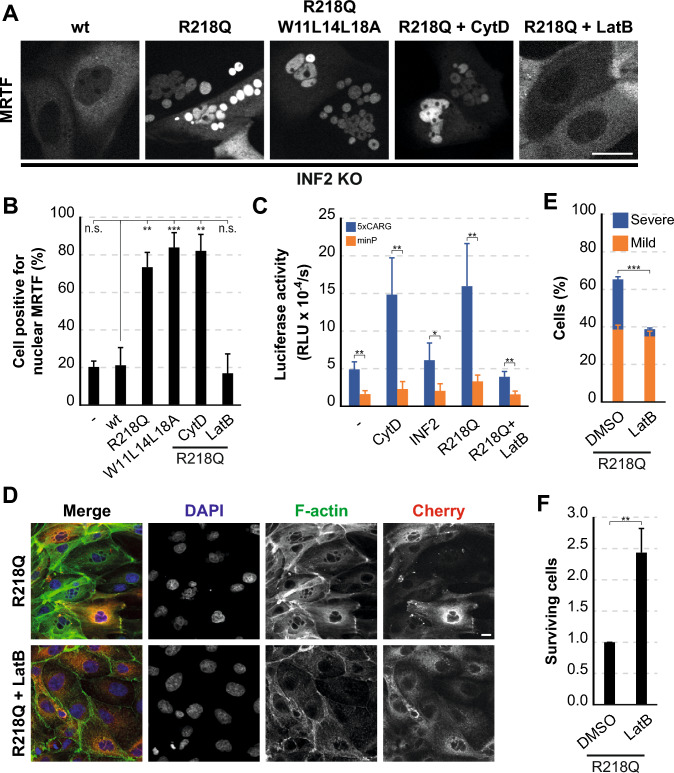
INF2 R218Q induces entry of MRTF into the nucleus and activation of the MRTF-SRF transcriptional complex. **A** Images of MRTF-GFP distribution in wt INF2, INF2 R218Q and INF2 R218Q W11L14L18A INF2 KO MDCK cells. Cells were treated or untreated with 100 nM CytD or 100 nM LatB as indicated. **B** Percentage of cells with MRTF-GFP in the nucleus. More than 300 cells were examined; three independent experiments. **C** Luciferase reporter gene activity from MDCK cells transfected with plasmids containing the luciferase gene with a minimal promoter without (minP) and with five canonical CArG boxes (5xCArG). Luciferase activity was measured after 24 h of exogenous INF2 expression. For INF2 R218Q, cells were left untreated or treated with LatB for the last 17 h. As a positive control of luciferase activity, we used cells treated with 500 nM CytD for the last 4 h. Three independent experiments were performed. **D** Image of an equatorial plane of MDCK cells expressing INF2 R218Q cells for 48 h that were treated or not with 100 nM LatB for the last 24 h. Nuclei were visualized with DAPI. **E** Percentage of cells displaying mild or severe nuclear phenotypes. More than 300 cells were examined; three independent experiments*.* **F** Percentage of surviving cells*.* Over 1200 cells were examined; three independent experiments*.* DMSO was used as vehicle. Scale bars, 10 μm. n.s., not significant; *, *p* < 0.05; **, *p* < 0.01; ***, *p* < 0.001

Members of the MRTF family, comprising MRTF-A and B (referred to simply as MRTF), form a complex with the serum response factor (SRF), a widely expressed mammalian transcription factor, modulating its activity [28, 56]. MRTF associates with G-actin, maintaining an inactive state in the cytosol. When cytosolic G-actin concentration decreases due to massive actin polymerization, G-actin-free MRTF translocates into the nucleus. There, it associates with SRF to direct gene transcription [57]. In MDCK and other cell lines, MRTF relocation to the nucleus responds to increased Ca^2+^ levels in an INF2 expression-dependent manner [13]. This observation indicates that massive actin polymerization by Ca^2+^/CaM-activated INF2 depletes G-actin from the cytosol, facilitating MRTF’s nuclear translocation. The importance of INF2 in localizing MRTF is also evidenced by the observation that whereas MRTF is partly nuclear at steady state in normal cells, it is mainly cytosolic in INF2 knockdown HeLa cells [13], and almost totally cytosolic in INF2 KO RPE-1 cells [51] and INF2 KO MDCK cells [12]. Remarkably, INF2 R218Q, but not wt INF2, facilitated MRTF nuclear entry in INF2 KO MDCK cells without Ca^2+^ signaling, even in the absence of a functional CaM- and centrin-binding site (Fig. 8A, B). These findings indicate that the constitutive actin polymerization activity of INF2 R218Q is sufficient to drive the nuclear translocation of MRTF.

Cytochalasin D (CytD) and latrunculin B (LatB) are natural compounds that produce F-actin disassembly of F-actin through distinct mechanisms [58]. Beyond their impact on F-actin, CytD disrupts the actin-MRTF complex, liberating MRTF and facilitating its translocation to the nucleus. In contrast, LatB stabilizes this complex, retaining MRTF in the cytosol [28, 59]. We treated INF2 R218Q cells with CytD and LatB at concentrations that were suboptimal to block F-actin formation completely, as otherwise the formation of the cleavage furrow and the actin cytokinetic ring, and consequently, the process of cytokinesis would be affected. Consistent with its effect on the actin-MRTF complex, CytD treatment did not alter the proportion of INF2 R218Q cells with nuclear MRTF, which was already notably high (Fig. 8A, B). As expected, LatB treatment significantly reduced the presence of MRTF within the nucleus of INF2 R218Q cells (Fig. 8A, B). To directly assess the effect of INF2 R218Q on MRTF-SRF-mediated transcription, we employed a luciferase reporter plasmid containing five canonical MRTF-SRF responsive elements (5xCArG) upstream of a minimal promoter [60]. In line with the effect of INF2 R218Q on MRTF nuclear translocation, INF2 R218Q caused a considerable increase of MRTF-SRF transcriptional activity, an effect that was reduced by LatB (Fig. 8C).

Suggesting the involvement of MRTF-SRF activation in the detrimental effect of pathogenic INF2 on cells, our findings revealed that LatB significantly reduced the percentage of cells with a severe nuclear phenotype while leaving the count of mild nuclear phenotype cells unchanged (Fig. 8D, E). One plausible explanation for this distinct effect is that attaining a severe nuclear phenotype requires a more pronounced activation of MRTF-SRF than to achieve a milder phenotype. Since LatB only partially inhibited MRTF-SRF activity, our experimental conditions, its impact might suffice to shift cells that would otherwise display a severe phenotype toward a milder one, and possibly ameliorates the nuclear defects in cells that, without LatB, would exhibit mild nuclear abnormalities. It is of note that, consistent with the decrease in the number of cells with nuclear abnormalities, LatB increased the number of surviving INF2 R218Q cells (Fig. 8F).

### Pathogenic INF2 drives extensive transcriptome reprogramming

**Fig. 9:**
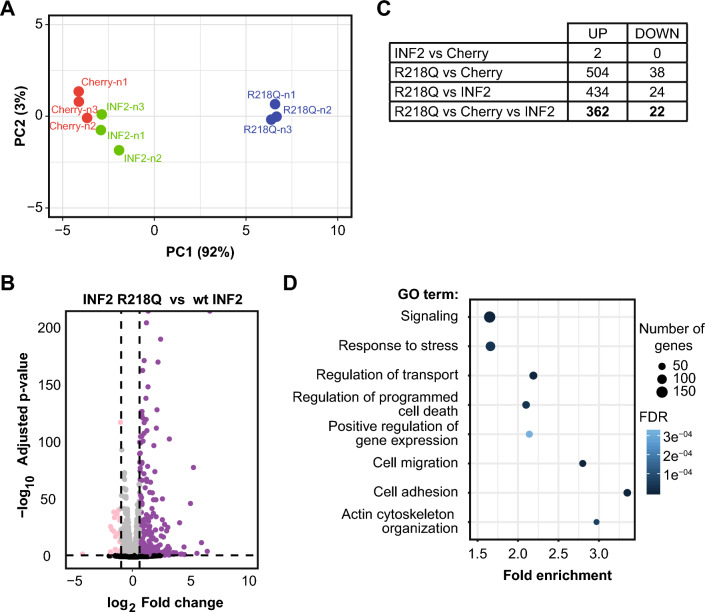
INF2 R218Q-induced transcriptome reprogramming*.* **A** Principal component analysis illustrating intragroup and intergroup variability across RNA-seq datasets from wt INF2, INF2 R218Q, and Cherry MDCK cells. **B** Volcano plot depicting significantly depleted (pink) and enriched (purple) mRNAs found in INF2 R218Q cells relative to wt INF2 cells. The discontinuous vertical lines indicate the thresholds used: 1.5-fold for upregulated and 0.5-fold for downregulated genes. The discontinuous horizontal line indicates the threshold of *p* < 0.05. Black, genes with *p* > 0.05. **C** Count of differentially expressed genes in INF2 R218Q cells with ≥ 50% difference of expression levels and *p* < 0.05. **D** Gene ontology analysis of the genes differentially expressed in INF2 R218Q cells. Only the 384 genes selected in (C) were considered

To gain a comprehensive understanding of the effect of pathogenic INF2 on transcription, we conducted RNA sequencing analysis, comparing INF2 R218Q cells with wt INF2 and Cherry cells, which served as control cells. Principal component and correlation analyses revealed minimal intragroup and marked intergroup differences (Figs. 9A and S5A). The pairwise comparison of differential gene expression between INF2 R218Q, wt INF2, and Cherry cells are illustrated through volcano plots (Figs. 9B and S5B, C). Subsequent examination identified 1345 genes upregulated and 1039 downregulated in INF2 R218Q cells compared simultaneously with both types of control cell, statistical significance being concluded for values of *p* < 0.05 (Fig. S5D). This result implies a profound restructuring of the transcriptome induced by pathogenic INF2. To narrow our focus to genes with a more substantial difference in expression, we applied a threshold of a 50% change in expression levels. This led to a reduced list of 384 genes, with 362 being upregulated and 22 downregulated in INF2 R218Q cells. Notably, a minimal difference in the transcriptome was observed between wt INF2 cells and Cherry cells (Fig. 9C). Among the refined list of 362 upregulated genes in INF2 R218Q cells, 32 were recognized as targets of the MRTF-SRF complex [61], while 47 genes were associated with p53 regulation [39] (Tables S1-8). Gene ontology enrichment analysis of the 384 genes highlighted significant associations with the actin cytoskeleton, cell death, cell adhesion, cell migration, response to stress, signaling and regulation of gene expression, and transport (Fig. 9D).

In summary, INF2 R218Q induces a fundamental remodeling of the transcriptome, implying that it has a profound impact on multiple cellular processes.

### LatB protects human primary podocytes from INF2 R218Q-induced challenges

**Fig. 10:**
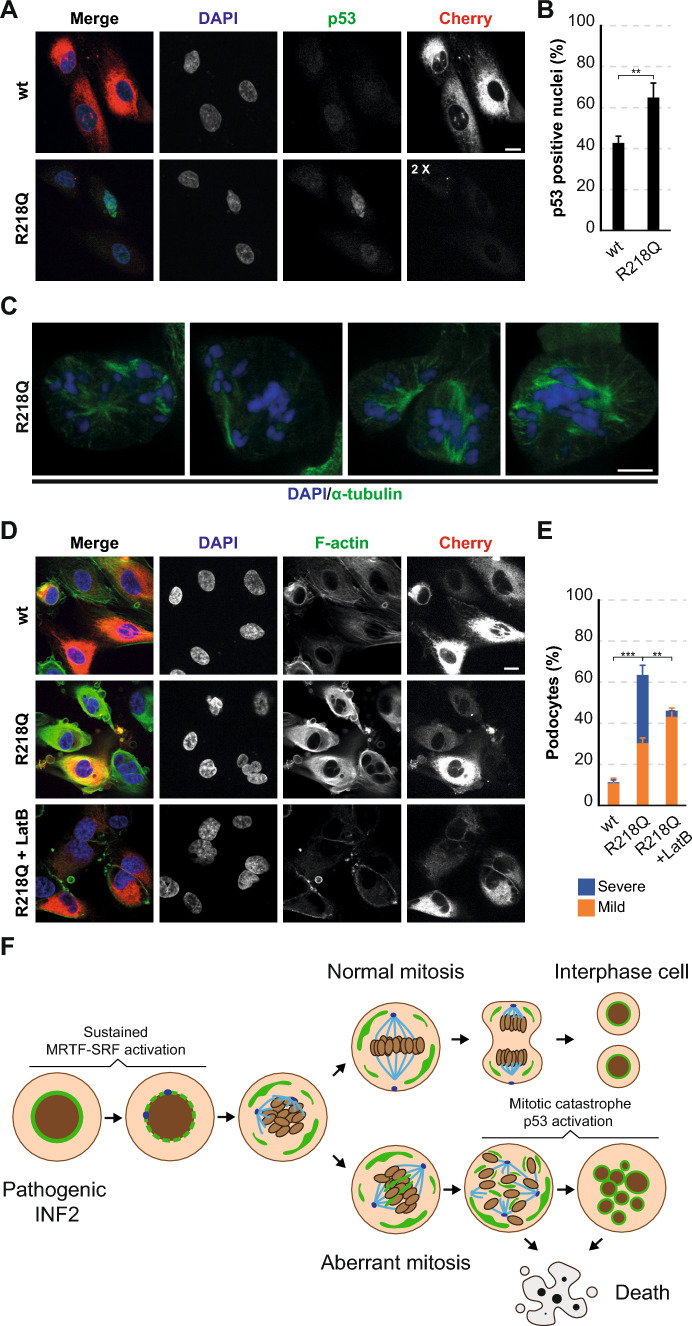
Effect of INF2 R218Q in human primary podocytes. **A** Images of podocytes expressing wt INF2 and INF2 R218Q stained for p53. **B** Percentage of cells positive for nuclear p53. **C** Images of INF2 R218Q mitotic podocytes displaying abnormal spindles. Cells were stained for α-tubulin. Chromosomes were visualized using DAPI. **D** Images of podocytes expressing wt INF2 or INF2 R218Q for 48 h that were treated or not with 100 nM LatB for 24 h. Cells were stained for F-actin. **E** Percentage of cells displaying nuclear abnormalities. More than 300 cells were examined; three independent experiments. Nuclei were visualized with DAPI. Scale bars, 10 μm (**A**, **D**), 5 μm (**C**). **, *p* < 0.01; ***, *p* < 0.001. **F** Schematic depicting the effect of pathogenic INF2. Abnormal mitosis leads to cell death during mitosis or results in cells with nuclear abnormalities that eventually die. The ER is represented in green, chromosomes in brown, and microtubules in blue

To validate some of our main observations in a more physiological cell model system, we examined the effect of INF2 R218Q in human primary podocytes. Remarkably, expression of INF2 R218Q induced p53 translocation to the nucleus (Fig. 10A, B) and resulted in abnormal mitotic spindles and aberrant nuclei (Fig. 10C–E), mirroring the effects observed in MDCK cells. Notably, as in the latter cells, LatB treatment produced partial mitigation of the abnormal nuclear phenotype (Fig. 10D, E).

## Discussion

The progressive loss of podocytes leads to FSGS which, depending on the degree of glomerular damage, exhibits a spectrum of clinical manifestations from proteinuria to nephrotic syndrome or chronic kidney disease, culminating in end-stage renal disease [1]. Heterozygous mutations of *INF2* are associated with an inherited form of FSGS (FSGS5; OMIM: 613237) that typically emerges in adolescence or adulthood [4, 31]. Our investigation revealed that pathogenic INF2 expression disrupts normal mitosis by causing the appearance of multiple MTOCs that assemble multipolar spindles, hampering chromosome alignment at the metaphase plate. The space occupied by the spindles is invaded by the ER, which envelops the scattered chromosomes, forming abnormal nuclei, predominantly multiple micronuclei. Cells undergo mitotic catastrophe within a few days and die. A mutation in the catalytic domain inactivating INF2 prevents nuclear defects, while mutations that activate INF2 mimic the observed nuclear abnormalities. Pathogenic INF2 induces nuclear translocation of the transcriptional cofactor MRTF, profoundly altering gene transcription through MRTF-SRF activation. Pharmacological inhibition of MRTF nuclear translocation significantly reduces multi-micronucleation and cell death. The effects described here could be similar to those that occasionally occur in podocytes of patients with INF2-linked FSGS, opening up potential strategies for impeding disease progression.

Distinct INF2 variants display different effects: some result solely in FSGS, while others lead to FSGS alongside CMT. This suggests that podocytes being more susceptible than Schwann cells to pathogenic INF2. Moreover, renal symptoms manifest earlier in patients with variants causing both conditions as those in variants causing only FSGS. These observations are consistent with in silico predictions that suggest a greater degree of conformational alterations in the DID of variants causing the dual disease, thereby intensifying deregulation and cellular damage [24]. Despite this, our assay of nuclear abnormality formation in MDCK cells revealed no clear differences between pathogenic INF2 variants, except for INF2 L76P. This lack of distinction may be attributed to the tendency for exogenous expression to equalize the cell damage produced. The milder effect observed with INF2 L76P and INF2 3LA on F-actin levels and nuclear morphology compared with INF2 R218Q could be due to residual regulation of their activity. Since the expression of natural benign INF2 variants had no nuclear effects, a potential application of our findings is to use of the formation of multinucleated MDCK cells as an in vitro test to differentiate pathogenic from benign INF2 variants.

In accordance with previous studies emphasizing constitutive actin polymerization activity [11, 26], our investigation revealed higher levels of F-actin in cells expressing pathogenic INF2 compared to those expressing exogenous wt INF2, despite the former’s lower expression levels. These effects of pathogenic INF2 underscored its gain-of-function characteristics, as no alterations were detected in endogenous INF2 levels. Our RNA-sequencing analysis identified a large number of genes in INF2 R218Q cells whose expression levels differed by at least a 50% compared with wt INF2 and Cherry cells. A notable proportion of these genes are established targets of the actin-MRTF-SRF transcriptional circuit [61], which is acknowledged for its role in regulating the cytoskeleton, transcription, vesicular transport, and several other important cellular processes. Additionally, we identified multiple genes associated with cell death that are potentially regulated by p53 [39]. Given the pivotal role of INF2 R218Q’s actin polymerization activity in the emergence of nuclear abnormalities, it appears that sustained activation of the actin-MRTF-SRF transcriptional circuit is the primary driver of the cellular alterations underlying mitotic chaos, ultimately leading to cell death via p53-dependent activation (Fig. 10F). Supporting this interpretation, our findings revealed that stabilizing the actin-MRTF complex using a suboptimal concentration of LatB significantly mitigated the emergence of nuclear abnormalities and subsequent cell death.

Pathogenic INF2 induced pronounced nuclear abnormalities in non-transformed cell lines (MDCK, LLC-PK1, RPE-1, and PtK2), contrasting with the considerably milder or negligible impact observed in transformed cell lines (HEK293T and HeLa). This disparity may be attributed to the inhibition of p53 by the SV40 large T-antigen in HEK293T cells and p53 degradation mediated by the human papillomavirus protein E6 in the case of HeLa cells, as reported in previous studies [62, 63]. This suggests p53-independent protective mechanisms against pathogenic INF2, possibly establishing diverse damage thresholds among cell types. Podocytes, parietal epithelial cells (PECs), and tubular epithelial cells share common origins during nephrogenesis, displaying similar transcriptomes [64–66]. Among the non-transformed cells assayed, INF2 R218Q had the most significant impact on nuclear morphology in renal MDCK cells, which derive from distal tubules, and LLC-PK1 cells, which derive from proximal tubules [32, 67]. Consequently, MDCK cells could be considered a cell model not distantly related to podocytes and PECs. Accordingly, it was not surprising that human primary podocytes recapitulated the main results obtained in MDCK cells.

Silencing INF2 in zebrafish causes a spectrum of developmental anomalies, including embryo malformation, impaired nephrogenesis, slit diaphragm deformities, and mislocalization of nephrin [68]. In primary podocytes from INF2 R218Q knock-in mice, the interaction between dynein light chain 1 and INF2 is disrupted, resulting in altered dynein-mediated post-endocytic sorting of nephrin to lysosomes and subsequent degradation [69]. Notably, despite these in vitro molecular disruptions, INF2 R218Q knock-in mice do not exhibit renal pathology, suggesting an intact kidney developmental process. Nevertheless, podocyte plasticity is impaired in these mice, hindering the restoration of typical podocyte architecture following acute kidney injury [70]. The absence of overt FSGS in these mice may result from inherent differences between human and mouse podocytes or from the possible requirement of repeated kidney injuries over time for FSGS to be manifested.

Throughout the human lifespan, podocyte loss occurs when damaged podocytes detach, creating small gaps in the glomerular basement membrane (GBM). These gaps prompt neighboring, undamaged podocytes to attempt division to fill the void. Despite being post-mitotic cells, adult podocytes might still have limited potential for cell division to give rise to two new podocytes to mend GBM gaps [71, 72]. However, failed attempts to divide result in larger denuded areas. Recent RNA-sequencing identified specific PECs as putative reservoirs of podocytes [73]. During development and in young mice, podocyte renewal occurs through PEC migration and trans-differentiation [74]. However, whether PECs serve as podocyte progenitors in adolescent and adult kidneys continuous to be debated [75]. Although our study was performed enterely in vitro, we cannot avoid speculating about the potential in vivo significance of our findings. It is plausible that the alteration of polarized trafficking by pathogenic INF2 [68, 69] leads to podocyte depolarization. These cells may either die directly undergo mitotic catastrophe in their attempt to divide. If normal repair of denuded GBM gaps occurs through adjacent podocyte division, PEC proliferation and differentiation, or both, pathogenic INF2 would lead to mitotic catastrophe, hindering glomerular regeneration, and thereby favoring gradual FSGS progression in humans.

FSGS consistently results in podocyte loss [1, 76] and is often associated with mitotic catastrophe as the primary mechanism of podocyte death [77, 78]. Multinucleated cells, found in the urine and glomeruli of FSGS patients, indicate non-viable cells that probably detach from the glomeruli [79–85]. Our results highlight how in INF2-linked FSGS, pathogenic INF2 sustainably activates the MRTF-SRF complex's transcriptional activity, extensively reprogramming the cell transcriptome. Subsequently, this process causes multifactorial mitosis failure, multinucleation, and other nuclear abnormalities leading to mitotic catastrophe-mediated cell death, followed by cell detachment. Further experimentation is needed to determine whether the effects of pathogenic INF2 observed in cultured cells also occur in animal models. The activation of the MRTF-SRF complex by pathogenic INF2 is consistent with our proposal that INF2 is a sensor that controls the levels of free G-actin to fine-tune MRTF-SRF complex-mediated transcription in specific cell types [86]. The development of non-toxic pharmacological inhibitors targeting the actin-MRTF-SRF circuit to modulate its transcriptional activity offers potential therapeutic avenues to mitigate INF2-related FSGS progression.

## Materials and methods

### Cells and cell culture

Canine epithelial MDCK II (# CRL-2936), human epithelial HEK293T (# CRL-3216), human epithelial HeLa (# CCL-2), human epithelial hTERT RPE-1 (# CRL-4000), porcine epithelial LLC-PK1 (# CRL-1642) and rat kangaroo epithelial PtK2 (# CCL-56) cells were obtained from the ATCC. MDCK, PtK2 and HeLa cells were grown in MEM (Gibco, #61100) supplemented with 5–10% fetal bovine serum (FBS, Gibco, #A4766801), while HEK293T and LLC-PK1 cells, and RPE-1 cells were maintained in DMEM (Gibco, #52100) or DMEM/F12 (Gibco, #32500), respectively, supplemented with 10% FBS. The INF2 KO MDCK cell clone [52] and the INF2 KO MDCK cells stably expressing GFP-MRTF-A [12] have been described previously. Human primary podocytes (Innoprot, # P10669) were grown on poly-L-lysine coated dishes using epithelial cell medium (Innoprot, #P60106) with 2% FBS. All cells were incubated at 37 °C in a 95% air/5% CO_2_ atmosphere. Routine mycoplasma testing was conducted to ensure cell-line integrity.

### Reagents and antibodies

The rabbit polyclonal antibody to INF2 has been described previously [17, 18]. The following primary commercial antibodies were used: rabbit polyclonal antibody to mCherry (Abcam # ab167453, WB 1:1000, IF 1:250), mouse mAb to GFP (Roche, # 11814460001, 1:1000), mouse mAb to GAPDH (Invitrogen, # AM4300, 1:10000), rabbit mAb to cleaved caspase-3 (Cell Signaling, # 9664, 1:1000), rabbit mAb to Ki67 (Sigma-Aldrich, # 275R-1, 1:1000), rabbit polyclonal antibody to centrin (Proteintech, # 12794-1-AP, 1:100), mouse mAb to γ-tubulin (Merck, # T6557, 1:1000), mouse mAb to total α-tubulin (ThermoFisher, # 62204, WB 1:5000, IF 1:1000), mouse mAb to acetylated α-tubulin (Merck, # T7451, 1:10000), rat mAb to tyrosinated α-tubulin (Merck, # MAB1864, WB 1:1000, IF 1:250), rabbit polyclonal antibody to detyrosinated α-tubulin (Merck, # AB3201, 1:1000), mouse mAb to lamin A/C (Santa Cruz, # sc-7292, 1:1000), mouse mAb to p53 (Proteintech, # 60283-2-Ig, WB 1/5000, IF 1/400), and mouse mAb to p21 (Santa Cruz, #sc-6246, 1/200). Secondary antibodies for IF were: donkey anti-rabbit IgG Alexa Fluor 488-conjugated antibody (ThermoFisher, # A-21206, 1:500), donkey anti-mouse IgG Alexa Fluor 488-conjugated antibody (ThermoFisher, #A-21202, 1:500), donkey anti-rabbit IgG Alexa Fluor 555-conjugated antibody (ThermoFisher, #A-31572, 1:500), goat anti-rat IgG Alexa Fluor 555-conjugated antibody (ThermoFisher, # A-21434, 1:500), and donkey anti-mouse IgG Alexa Fluor 647-conjugated antibody (ThermoFisher, # A-31571, 1:500). For WB the following HRP-conjugated antibodies were used: anti-rabbit IgG (GE Healthcare, # NA934, 1:5000), anti-mouse IgG (Jackson Immunoresearch, # 715-035-151, 1:5000), and anti-rat IgG (Invitrogen # 31470, 1:5000). CytD (# C8273) and LatB (# 428020) were from Merck, and roscovitine (# sc-24002) was from Santa Cruz.

### Molecular cloning

The p33Cherry vector [12] was used to express wt INF2-1 and wt INF2-2 as mCherry fusions. The pathogenic L57P, L76P, L77P, R106P, L128P, L165P and INF2 R218Q variants, the benign INF2 R212C, R261G and V194N natural variants, and the in silico designed INF2 mutants A149D, I643A and L976L977L986A were generated in p33Cherry-INF2-1 introducing point mutations using oligonucleotides with the appropriate substitutions made with the Quick-Change II directed mutagenesis kit (Stratagene, #200523). For some of the INF2 constructs, GFP fusions were prepared by substituting the Cherry coding sequence in p33Cherry with EGFP from the pEGFP-C1 vector, resulting in the p33GFP constructs. The INF2-2 R218Q construct was generated by replacing the N-terminal 800 bp EcoR I-Sal I fragment of INF2-2 by that of INF2-1 R218Q. DNA constructs in a modified pGEX-4 T-1vector were used to express GST fusions of INF2 fragments in *E. coli* for the pull-down assays. The pGFP-MRTF-A DNA construct was described [51]. For INF2 expression under the late CMV promoter, the Cherry-INF2 DNA fragment was cloned from the p33Cherry INF2 and INF2 R218Q constructs into the pCR3.1 vector (Promega). The DNA constructs expressing GFP fused to centrin-1 (# 72641), centrin-2 (# 41147), and centrin-3 (# 69746), CaM (# 47602), H2B (# 11680), sec61β (# 62008), HEC1 (# 114049) and α-tubulin (# 58197) were obtained from Addgene. For expression, the centrin-3 insert was cloned into the pEGFP-C1 vector. To enhance the expression of HEC1-GFP, the HEC1 coding sequences were cloned into the retroviral p33GFP vector. All constructs were validated through DNA sequencing (Macrogen).

### Exogenous INF2 expression

HEK293T cells in a p100 culture dish were cotransfected with retroviral plasmid DNA along with MLV-GagPol/pHIV 8.1 and pHIT VSVg plasmids (1.4 μg/ml) in a 4.6:3.3:1 ratio using polyethyleneimine (Polysciences, #23966). After 72 h, the supernatant containing the retroviral particles was harvested and filtered. For exogenous protein expression using infection, cells were treated with 10 μg/ml polybrene (Merck, # TR-1003-G) at 37 °C for 15 min and subsequently exposed to the supernatant containing the retroviral particles for 7 h. Post-incubation, cells were washed and incubated for the indicated times. Unless otherwise indicated, experiments were conducted 48 h after the onset of infection. For transient expression experiments, we used Lipofectamine 2000 following the manufacturer’s protocol (ThermoFisher, # 11668019).

### Transfections and generation of stable transfectants

MDCK cells stably expressing GFP-tubulin, GFP-sec61 and GFP-H2B were generated by transfection using Lipofectamine 2000. GFP-expressing single cells were FACS-sorted 48 h post-transfection and subsequently cultured under a selection pressure of 1 mg/ml G-418 for 14 days. The individual clones obtained from these transfections were screened using fluorescence microscopy and validated by immunoblotting using anti-GFP antibodies.

### Confocal microscopy and videomicroscopy

Cells were fixed with 10% formalin (37% formaldehyde solution; Merck, # HT501128) and permeabilized with 0.2% Triton X-100 or fixed and permeabilized with cold methanol for 5 min on ice. Subsequently, cells were blocked with 3% (wt/vol) BSA for 30 min, followed by successive incubation with the indicated primary antibody and the appropriate fluorescent secondary antibodies. Cells were also stained with DAPI (Merck, # 268298) and Alexa Fluor™ 488 phalloidin (ThermoFisher, # A12379) as indicated. After extensive washing, coverslips were mounted on glass slides with Fluoromount (Merck, #F4680). Imaging was conducted using a Nikon A1R + confocal laser-scanning microscope with a 60x (NA 1.2) water objective or an Olympus Spinning Disk Spin SR10 with 60x (NA 1.3) or 100x (NA 1.45) silicone immersion objectives. Resulting LSM images were converted to TIFF format. The perinuclear signal intensity was quantified using a ROI consisting of a 0.5-μm-width ring around the nucleus. The data represented correspond to the average intensity of three planes. For live-cell imaging, cells grown on µ-Slide 8 well chambers (Ibidi #80826) or a 35-mm glass-bottom dish (Ibidi, # 81218-200) were maintained at 37 °C in a 95% air/5% CO_2_ atmosphere in MEM without phenol red supplemented with 5% (v/v) FBS. The fluorophore silicon rhodamine (SiR) emits in far-red, thereby making it compatible with the emission of the GFP and Cherry proteins. SiR-DNA (Spirochrome, #SC007) and SiR-actin (Spirochrome, #SC001) were added to the cells 1 h before recording. Imaging was conducted with an Olympus Spinning Disk Spin SR10 with a 100x (NA 1.45) oil immersion objective or an sCMOS camera with a 40x (NA 1.2) water objective for extended recordings. Brightness and contrast adjustments, quantification of signal intensity, and measurement of cell area and circularity (defined as 4πA/L^2^, where A stands for the area and L for the perimeter) were carried out on an equatorial plane using Fiji software (https://fiji.sc).

### GST pull-down assay

To conduct pull-down assays, GST-fused proteins were expressed in *E. coli* BL21 cells (Stratagene, # 200131). Cultures were grown in LB media at 37 °C until an OD_600_ of 0.6–0.8 was reached. Subsequently, the temperature was lowered to 20 °C, and recombinant protein expression was induced by adding 0.5 mM IPTG (Apollo Scientific, # BIMB1008) for 16 h. Cell lysates from HEK293T cells expressing GFP alone or GFP-centrins 1–3 or GFP-CaM were incubated with assay buffer (20 mM Tris–HCl, pH 7.8, 150 mM NaCl, 1% Nonidet P-40, 1% glycerol, 1 mM sodium orthovanadate, 0.1 mM PMSF, and 5 mM CaCl_2_), supplemented with a commercial cocktail of protease inhibitors (Merck, # 11697498001) at 4 °C, alongside 30 µg of the indicated GST-fused proteins immobilized on glutathione-Sepharose 4B beads (Merck, # GE17-0756-01). Following a 3-h incubation, beads were collected by microfuge centrifugation and washed twice with cold PBS. Samples were analyzed by immunoblotting. Additionally, Coomassie blue staining was conducted on a separate aliquot of glutathione-Sepharose bead-immobilized GST-fused protein to control for the amount of GST protein used.

### Immunoblotting

After blocking with 5% BSA (w/v) and 0.05% (v/v) Tween-20 in Tris-buffered saline, membranes were incubated overnight with the indicated specific primary antibodies, washed with Tris-buffered saline containing 0.05% Tween 20, and incubated for 30 min with the appropriate secondary antibodies conjugated to HRP. Signals were visualized using Clarity Western ECL substrate (BioRad, # 1705060).

### Transcription reporter assay

The pGL4.3.4[luc2P]/SRF-RE/Hygro vector (Promega, # E1350) features five consensus CArG boxes interspersed with nucleotide spacers, followed by a minimal promoter located upstream of a modified firefly luciferase gene (5xCArG plasmid). A derivative of this vector was generated by removing the CArG boxes resulting in the minP plasmid. DNA constructs (180 ng) were co-transfected with 20 ng of the pRL-TK Renilla luciferase reporter plasmid (Promega, # E2241) using Lipofectamine 2000. After 24 h, for INF2 expression, the cells were infected with recombinant retroviral particle supernatants for 7 h, washed, and then incubated at 37 °C for 17 h in culture medium with 1% FBS. Luminescence was measured using the FluoStar Microplate Reader equipment (BMG Labtech) and the Dual-Luciferase reporter assay system (Promega, # E1910).

### RNA sequencing and data analysis

Libraries were prepared using the TruSeq stranded mRNA Library Prep protocol (Illumina, # 20020595). This process converts total RNA into a library of template molecules with known strand origin, suitable for subsequent cluster generation and DNA sequencing. Initially, 1000 or 500 ng of total RNA underwent polyA-mRNA selection using Oligo-dT beads, with two rounds of purification. During the second elution, mRNA was fragmented at an elevated temperature and primed with random hexamers for cDNA synthesis, the latter being achieved by reverse transcriptase (SuperScript II; Invitrogen, # 18064-014). Second-strand cDNA was then synthesized, incorporating dUTP in place of dTTP, resulting in blunt-ended ds cDNA. A single ‘A’ nucleotide was added to the 3’ ends of the blunt, followed by ligation of Truseq adapters. Finally, PCR selectively enriched those DNA fragments that have adapters at both ends, utilizing Unique Dual Indexes and the provide kit master mix. Purification steps employed AgenCourt AMPure XP beads (Beckman Coulter, # A63882). Final libraries were analyzed using Bioanalyzer DNA 1000 or Fragment Analyzer Standard Sensitivity (Agilent, # 5067-1504 or DNF-473) for quantity estimation and size distribution validation. Quantification was performed by qPCR using the KAPA Library Quantification Kit KK4835 (Roche, # 07960204001). Sequencing was carried out on an Illumina NovaSeq6000 instrument. Quality analyses were performed over reads using FastQC (v0.11.8) software. The reads were aligned against the most recent *Canis familiaris* default reference genome (ROS_Cfam_1.0) using the Histat2 (v2.1.0) aligner (http://daehwankimlab.github.io/hisat2). The differential expression analysis was performed using Deseq2 (https://bioconductor.org/packages/release/bioc/html/DESeq2), and R software package (https://www.r-project.org). Gene ontology enrichment analysis was performed with AmiGo 2 (https://amigo.geneontology.org/amigo).

### Quantitative RT-PCR

Total RNA isolated from cultured cells were purified using the RNeasy (Qiagen, # 74104). The iScript cDNA Synthesis kit (Biorad PN170-8891) was utilized for cDNA synthesis, followed by qPCR using the Sso Fast EvaGreen Supermix (Biorad, CN 172-5204) on a CFX Opus 384 Real Time PCR System equipment (Bio-Rad). INF2 mRNA levels were normalized relative to those of GAPDH and tyrosine 3-monooxygenase/tryptophan 5-monooxygenase activation protein zeta (YWHAZ). Each experiment, performed in triplicate, was part of a series of between three independent trials. Data were analyzed with GenEX software. For information on the specific oligonucleotide primers used for qPCR, refer to Table S9.

### Statistical analysis

Graphical representations and statistical analyses were primarily conducted with RStudio, version 4.2.1 software (https://www.rstudio.com). Box plots show the median values. Statistical significance was estimated from Mann–Whitney–Wilcoxon tests. The other graphs show the mean values and SD, differences in them being tested by two-tailed Student’s unpaired t-tests. The significance levels are denoted as follows: n.s., not significant; *, *p* < 0.05; **, *p* < 0.01; ***, *p* < 0.001.

### Supplementary Information

Below is the link to the electronic supplementary material.

Supplementary file1 (PDF 4873 kb)

Supplementary file2 (DOCX 13 kb)

Supplementary file3 (XLSX 190 kb)

Supplementary file4 (AVI 5472 kb)

Supplementary file5 (AVI 12201 kb)

Supplementary file6 (AVI 5577 kb)

Supplementary file7 (AVI 16326 kb)

Supplementary file8 (AVI 4502 kb)

## Data Availability

The datasets generated in the current study are available from the corresponding author upon request. Sequencing raw data was submitted to the European Nucleotide Archive (https://www.ebi.ac.uk) with the accession number PRJEB71831.
