# Supplementary material for: INF2 formin variants linked to human inherited kidney disease reprogram the transcriptome, causing mitotic chaos and cell death
Source: Cell Mol Life Sci. 2024 Jun 25;81(1):279. doi: 10.1007/s00018-024-05323-y (PMC11335204; doi:10.1007/s00018-024-05323-y)
Supplement: Supplementary file 1 — Supplementary file1 (PDF 4873 kb) [file 18_2024_5323_MOESM1_ESM.pdf]

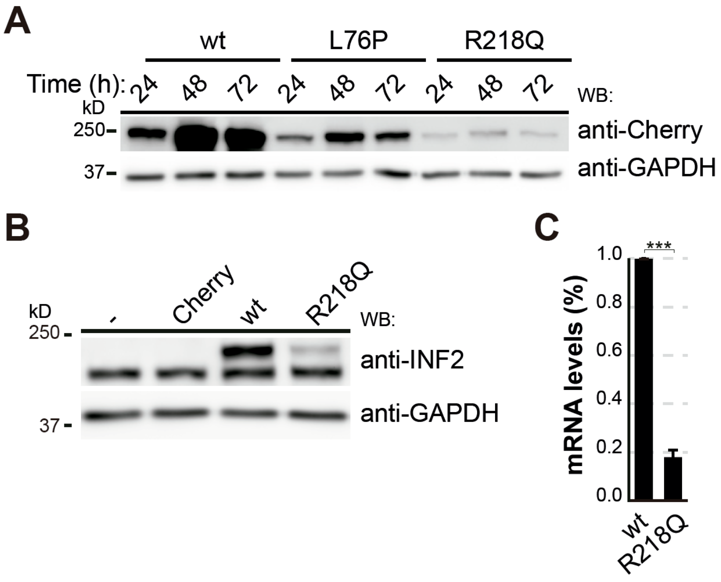

Fig S1 Expression of exogenous pathogenic INF2 in MDCK cells. (**A**) Time-course of expression of Cherry fusions of wt INF2 and the L76P and R218Q INF2 variants. Cell extracts were obtained at the indicated times of expression and were immunoblotted with anti-Cherry antibodies. Glyceraldehyde 3-phosphate dehydrogenase (GAPDH) expression served as a loading control. (**B**) Endogenous INF2 and exogenous wt INF2 and INF2 R218Q levels after 48 h of expression. Cell extracts were immunoblotted with anti-INF2 antibodies. (**C**) Transcript levels of exogenous wt INF2 and INF2 R218Q after 48 h of expression. The percentages of positive cells were considered for the estimations. Three independent experiments were performed. ***, $p < 0.001$.

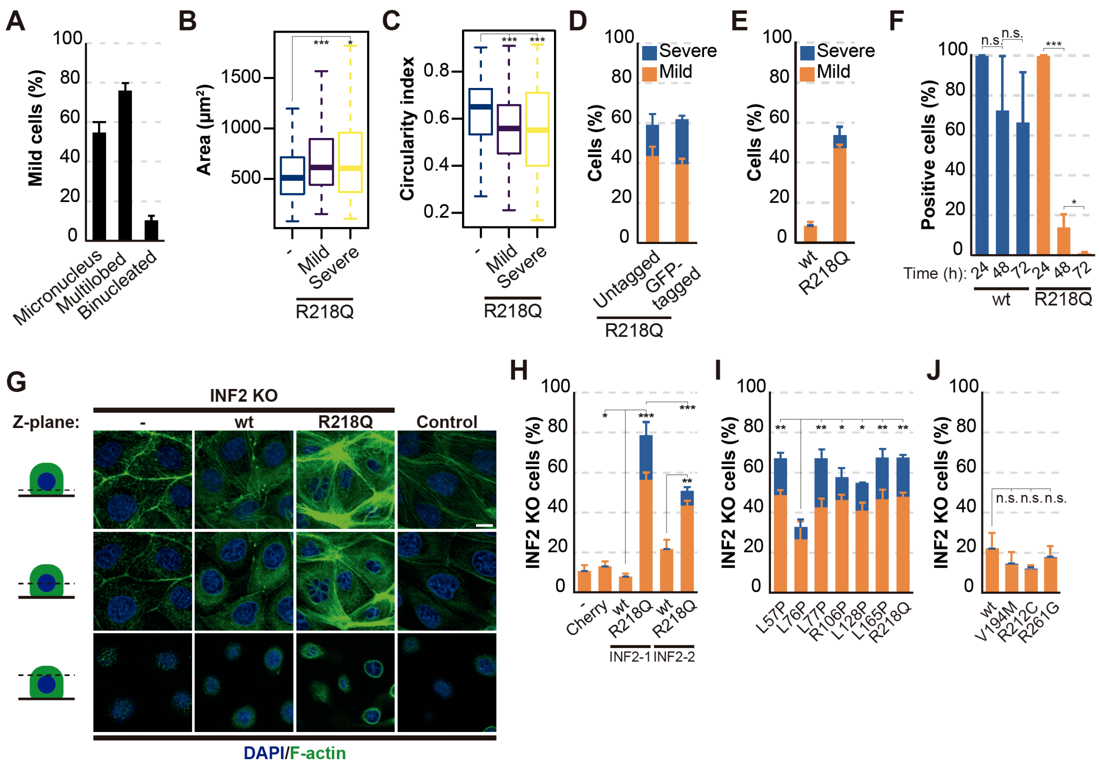

Fig S2 Other pathogenic INF2 variants also induce nuclear abnormalities. (**A**) Percentage of INF2 R218Q MDCK cells exhibiting different classes of "non-exclusive" abnormal nuclear morphologies. More than 200 cells displaying mild nuclear phenotypes were assessed across three independent experiments. (**B**) Analysis of cell area in control and INF2 R218Q cells. (**C**) Circularity index of control and INF2 R218Q cells. A value of 1.0 indicates a perfect circle. Decreasing circularity values indicate an increasingly elongated polygon. More than 140 cells were analyzed for each experimental condition in three independent experiments (B, C). (**D**) Assessment of nuclear phenotype in MDCK cells expressing either untagged or GFP-tagged INF2 R218Q. (**E, F**) Cells were transfected with plasmids expressing wt INF2 or INF2 R218Q under the control of the CMV promoter. Percentage of wt INF2 and INF2 R218Q MDCK cells exhibiting nuclear abnormalities after 48 h of transfection (E) and of cells positive for exogenous INF2 at the indicated times after transfection (F). Note that most of the cells were dead after 48 h and that most of the remaining cells had nuclear aberrations. (**G**) F-actin staining of control MDCK cells and INF2 KO MDCK cells expressing or not wt INF2 and INF2 R218Q. Bottom, equatorial and top planes are shown. Note the absence of the perinuclear F-actin ring in the equatorial plane of INF2 KO cells. Scale bar, 10 µm. (**H**) Percentage of INF2-1 R218Q and INF2-2 R218Q INF2 KO MDCK cells exhibiting mild or severe nuclear phenotypes. (**I, J**) Percentage of INF2 KO MDCK cells expressing the indicated pathogenic (I) and benign (J) INF2 variants exhibiting mild or severe nuclear phenotypes. More than 300 cells were analyzed for each experimental condition in three independent experiments in (D-F and H-J). n.s., not significant; *, $p < 0.05$; **, $p < 0.01$; ***, $p < 0.001$.

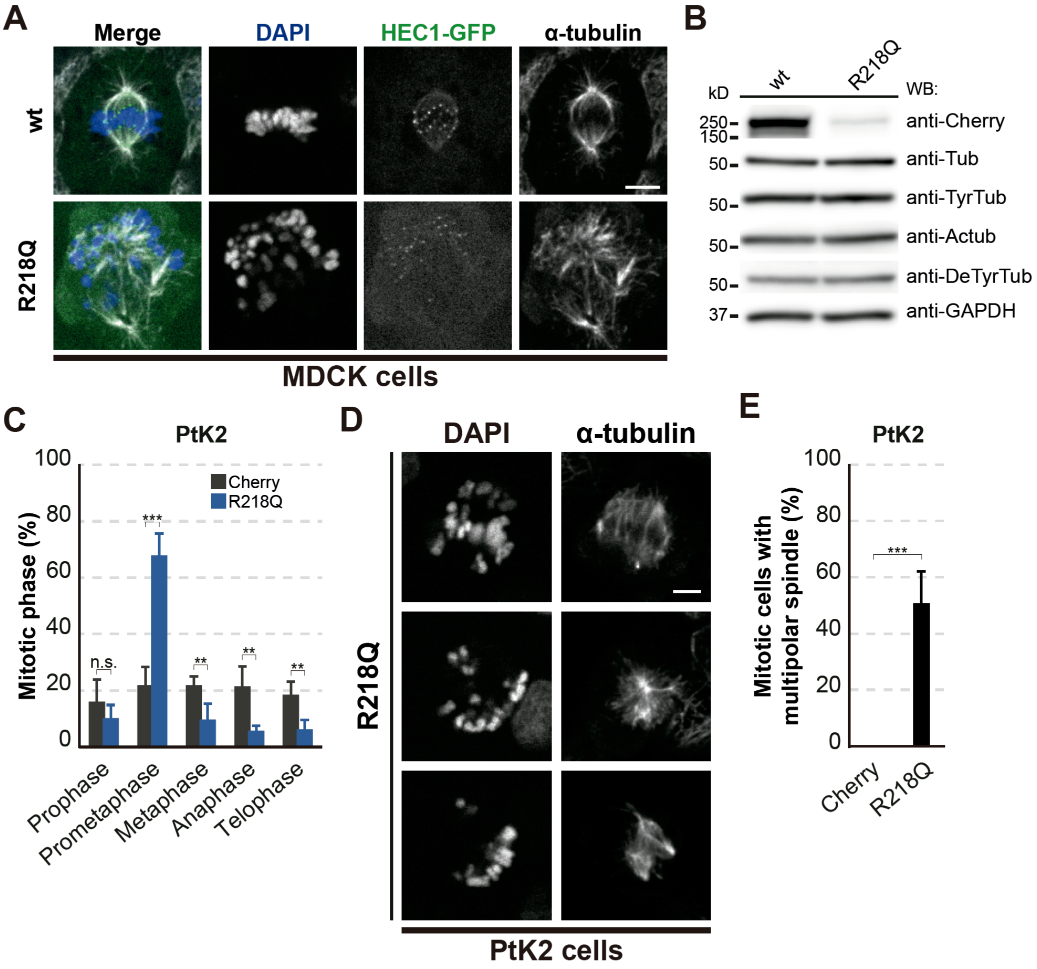

Fig S3 Effect of pathogenic INF2 on mitosis in PtK2 cells. (**A**) Images of wt INF2 and R218Q INF2 MDCK cells expressing HEC1-GFP during mitosis. Cells were stained for α-tubulin and with DAPI. (**B**) Immunoblot analysis of wt INF2 and INF2 R218Q MDCK cells using antibodies to Cherry, total α-tubulin, and the indicated posttranslational modifications of α-tubulin. GAPDH was used as a loading control. (**C**) Percentage of mitotic PtK2 cells expressing Cherry alone or fused to INF2 R218Q in the different phases of the cell cycle. More than 200 cells were examined; four independent experiments. (**D**) Images of mitotic INF2 R218Q PtK2 cells with multiple spindles. (**E**) Percentage of Cherry and INF2 R218Q PtK2 cells displaying multiple spindles. 45 Cherry cells and 136 INF2 R218Q cells were examined; four independent experiments. Scale bars, 5 µm. n.s., not significant; **, $p < 0.01$; ***, $p < 0.001$.

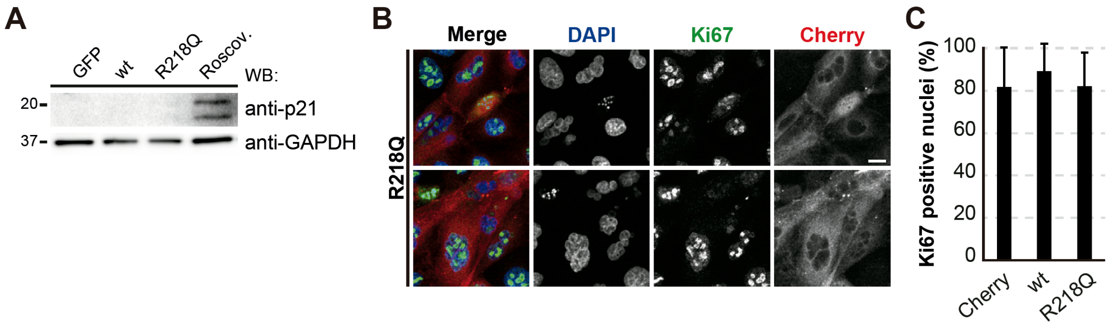

Fig S4 INF2 R218Q does not induce p21 expression. (**A**) Immunoblot analysis of p21 in GFP, wt INF2 and R218Q INF2 MDCK cells after 48 h of the expression of the exogenous proteins. A positive control of cells treated with 10 µg/ml roscovitine, an inhibitor of cyclin-dependent kinases, was included. GAPDH was used as a loading control. (**B**) Ki67 staining of INF2 R218Q MDCK cells after 48 h of INF2 R218Q expression. Nuclei were stained with DAPI. Two different cell fields are shown. Scale bar, 10 µm. (**C**) Percentage of Ki67-positive cells. Over 600 cells were examined per condition; three independent experiments.

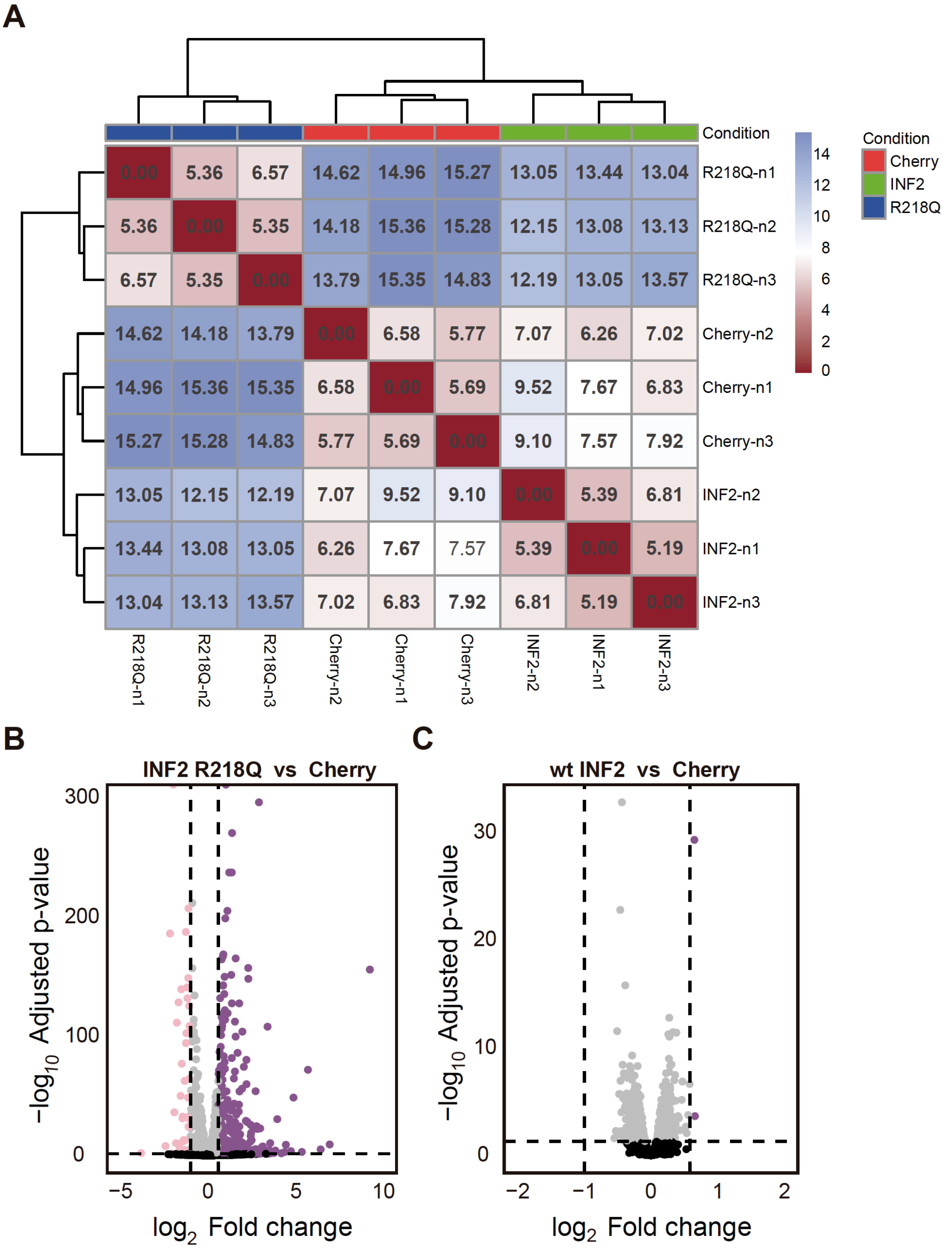

D

	UP	DOWN
INF2 vs Cherry	485	509
R218Q vs Cherry	2081	1946
R218Q vs INF2	1682	1481
R218Q vs Cherry vs INF2	**1345**	**1039**

Fig S5 INF2 R218Q-induced transcriptome reprogramming in MDCK cells. (**A**) Correlation analysis depicting relationships between various RNA-seq datasets. (**B**, **C**) Volcano plots displaying significantly depleted (pink) and enriched (purple) mRNAs in INF2 R218Q cells relative to Cherry cells (B), and in wt INF2 cells relative to Cherry cells (C). The discontinuous vertical lines indicate the thresholds used: 1.5-fold for the upregulated genes and 0.5-fold for the downregulated genes. The discontinuous horizontal line indicates the threshold of 0.05 used for the p-value. Black, genes with $p > 0.05$. (**D**) Total count of genes differentially expressed in INF2 R218Q cells relative to wt INF2 and Cherry cells ($p < 0.05$).
