# Supplementary material for: INF2 formin variants linked to human inherited kidney disease reprogram the transcriptome, causing mitotic chaos and cell death
Source: Cell Mol Life Sci. 2024 Jun 25;81(1):279. doi: 10.1007/s00018-024-05323-y (PMC11335204; doi:10.1007/s00018-024-05323-y)
Supplement: Supplementary file 2 — Supplementary file2 (DOCX 13 kb) [file 18_2024_5323_MOESM2_ESM.docx]

**Supplementary Tables**

**Table S1** Genes upregulated in wt INF2 cells relative to Cherry cells. Only genes with at least a 50% increased expression and a p-value < 0.05 were included.

**Table S2** Genes upregulated in INF2 R218Q cells relative to Cherry cells. Only genes with at least a 50% increased expression and a p-value < 0.05 were included.

**Table S3** Genes upregulated in INF2 218Q cells relative to wt INF2 cells. Only genes with at least a 50% increased expression and a p-value < 0.05 were included.

**Table S4** Genes upregulated in INF2 R218Q cells relative to both Cherry and wt INF2 cells. Only genes with at least a 50% increased expression and a p-value < 0.05 were included.

**Table S5** INF2 R218Q-upregulated genes that are potential targets of MRTF-SRF and/or p53. Only genes with at least a 50% increased expression relative to both Cherry and wt INF2 cells and a p-value < 0.05 were included.

**Table S6** Genes downregulated in INF2 R218Q cells relative to Cherry cells. Only genes with at least a 50% decreased expression and a p-value < 0.05 were considered.

**Table S7** Genes downregulated in INF2 R218Q cells relative to wt INF2 cells. Only genes with at least a 50% decreased expression and a p-value < 0.05 were considered.

**Table S8** Genes downregulated in INF2 R218Q cells relative to both Cherry and wt INF2 cells. Only genes with at least a 50% decreased expression and a p-value < 0.05 were considered.

**Table S9** Oligonucleotide primers used for qPCR analysis.

**Movie captions**

**Video** 1 ER invasion of the spindle zone in cells expressing pathogenic INF2. Cells expressing GFP-sec61β and INF2 L76P were stained with SiR-DNA. The arrowheads indicate regions of the mitotic spindle invaded by ER membranes.

**Video 2** ER, but not F-actin, invaded the spindle zone in cells expressing pathogenic INF2. Cells expressing GFP-sec61β and INF2 L76P were stained with SiR-actin. The arrowheads indicate regions of the mitotic spindle invaded by ER membranes.

**Video 3** Multinuclei formation in cells expressing pathogenic INF2. Cells expressing GFP-sec61β and Cherry-INF2 L76P were stained with SiR-DNA.

**Video 4** INF2 R218Q cells die and detach from the substrate. Cells expressing GFP-INF2 R218Q were stained with SiR-DNA. Differential interference contrast and fluorescence images of a cell dying and detaching during mitosis (left) or as a multinucleated cell (right). The arrowheads indicate the dying cells.

**Video 5** Dynamics of wt INF2 during mitosis. MDCK cells expressing GFP-tubulin and Cherry-wt INF2 were stained with SiR-DNA.
